# Author Correction: Imaging low-mass planets within the habitable zone of α Centauri

**DOI:** 10.1038/s41467-021-23145-5

**Published:** 2021-05-05

**Authors:** K. Wagner, A. Boehle, P. Pathak, M. Kasper, R. Arsenault, G. Jakob, U. Käufl, S. Leveratto, A.-L. Maire, E. Pantin, R. Siebenmorgen, G. Zins, O. Absil, N. Ageorges, D. Apai, A. Carlotti, É. Choquet, C. Delacroix, K. Dohlen, P. Duhoux, P. Forsberg, E. Fuenteseca, S. Gutruf, O. Guyon, E. Huby, D. Kampf, M. Karlsson, P. Kervella, J.-P. Kirchbauer, P. Klupar, J. Kolb, D. Mawet, M. N’Diaye, G. Orban de Xivry, S. P. Quanz, A. Reutlinger, G. Ruane, M. Riquelme, C. Soenke, M. Sterzik, A. Vigan, T. de Zeeuw

**Affiliations:** 1 Dept. of Astronomy and Steward Observatory, University of Arizona, Tucson, AZ USA; 2 NASA Nexus for Exoplanet System Science, Earths in Other Solar Systems Team, Tucson, AZ USA; 3 Institute for Particle Physics and Astrophysics, ETH Zurich, Zürich, Switzerland; 4 European Southern Observatory, Garching bei München, Germany; 5 STAR Institute, Université de Liège, Liège, Belgium; 6 AIM, CEA, CNRS, Université Paris-Saclay, Université Paris Diderot, Sorbonne Paris Cité, Gif-sur-Yvette, France; 7 Kampf Telescope Optics, München, Germany; 8 Lunar and Planetary Laboratory, University of Arizona, Tucson, AZ USA; 9 Univ. Grenoble Alpes, CNRS, IPAG, Grenoble, France; 10 Aix Marseille Univ, CNRS, CNES, LAM, Marseille, France; 11 Department of Materials Science and Engineering, Ångström Laboratory, Uppsala University, Uppsala, Sweden; 12 Subaru Telescope, National Astronomical Observatory of Japan, National Institutes of Natural Sciences (NINS), Hilo, HI USA; 13 The Breakthrough Initiatives, NASA Research Park, Moffett Field, CA USA; 14 James C. Wyant College of Optical Sciences, University of Arizona, Tucson, AZ USA; 15 LESIA, Observatoire de Paris, Meudon, France; 16 California Institute of Technology, Pasadena, CA USA; 17 Université Côte d’Azur, Observatoire de la Côte d’Azur, CNRS, Laboratoire Lagrange, Nice, France; 18 Jet Propulsion Laboratory, California Institute of Technology, Pasadena, CA USA; 19 Sterrewacht Leiden, Leiden University, Leiden, The Netherlands; 20 Max Planck Institute for Extraterrestrial Physics, Garching, Germany

**Keywords:** Exoplanets, Exoplanets

Correction to: *Nature Communications* 10.1038/s41467-021-21176-6, published online 10 February 2021.

The original version of this Article contained an error in Fig. 1b, in which the units incorrectly read ‘AU,’ instead of the correct ‘au’. This has been corrected in both the PDF and HTML versions of the Article.

The original version of this Article contained an error in Fig. 1b. In the original version of Fig. 1b, astronomical unit was considered instead of arc seconds, which results in a factor of about 1.3 in the values of ‘periastron’, ‘maximum stable orbit’ and the ‘semi-major axis (a)’ of the system. In the original version of Fig. 1b, ‘periastron’ incorrectly read ‘8.6 AU’ instead of the correct ’11.3 au’, ‘maximum stable orbit’ incorrectly read ‘~2.8 AU’ instead of the correct ‘~3.8 AU’, ‘semi major axis (a)’ incorrectly read ’17.6 ± 0.1 AU’ instead of the correct ‘23.6 ± 0.1 au’. This information is ancillary; the results are not affected. This has been corrected in both the PDF and HTML versions of the Article.

