# Supplementary material for: Imaging low-mass planets within the habitable zone of α Centauri
Source: Nat Commun. 2021 Feb 10;12:922. doi: 10.1038/s41467-021-21176-6 (PMC7876126; doi:10.1038/s41467-021-21176-6)
Supplement: Supplementary file 1 — Supplementary Information [file 41467_2021_21176_MOESM1_ESM.pdf]

Supplementary Information for

Imaging low-mass planets within the habitable zone of α Centauri

Authors: K. Wagner,[1, 2, *] A. Boehle,[3] P. Pathak,[4] M. Kasper,[4] R. Arsenault,[4] G. Jakob,[4] U. Käufl,[4] S. Leveratto,[4] A.-L. Maire,[5] E. Pantin,[6] R. Siebenmorgen,[4] G. Zins,[4] O. Absil,[5] N. Ageorges,[7] D. Apai,[1,2,8] A. Carlotti,[9] É. Choquet,[10] C. Delacroix,[5] K. Dohlen,[10] P. Duhoux,[4] P. Forsberg,[11] E. Fuenteseca,[4] S. Gutruf,[7] O. Guyon,[1, 12, 13] E. Huby,[14] D. Kampf,[7] M. Karlsson,[11] P. Kervella,[14] J.-P. Kirchbauer,[4] P. Klupar,[13] J. Kolb,[4] D. Mawet,[15] M. N'Diaye,[16] G. Orban de Xivry,[5] S. P. Quanz,[3] A. Reutlinger,[7] G. Ruane,[15, 17] M. Riquelme,[4] C. Soenke,[4] M. Sterzik,[4] A. Vigan,[10] and T. de Zeeuw[4, 18, 19]

[1] Dept. of Astronomy and Steward Observatory, University of Arizona, 933 N Cherry Ave, Tucson, AZ 85719, USA.

[2] NASA Nexus for Exoplanet System Science, Earths in Other Solar Systems Team.

[3] Institute for Particle Physics and Astrophysics, ETH Zurich, Wolfgang-Pauli-Strasse 27, 8093 Zürich, Switzerland.

[4] European Southern Observatory, Karl-Schwarzschild-Straße 2, 85748 Garching bei München, Germany.

[5] STAR Institute, Université de Liège, Allée du Six Août 19c, B-4000 Liège, Belgium.

[6] AIM, CEA, CNRS, Université Paris-Saclay, Université Paris Diderot, Sorbonne Paris Cité, F-91191 Gif-sur-Yvette, France.

[7] Kampf Telescope Optics, Alois-Gilg-Weg 7, 81373 München, Germany.

[8] Lunar and Planetary Laboratory, University of Arizona, 1629 E University Blvd, Tucson, AZ 85721, USA.

[9] Univ. Grenoble Alpes, CNRS, IPAG, 38000 Grenoble, France.

[10] Aix Marseille Univ, CNRS, CNES, LAM, Marseille, France.

[11] Department of Materials Science and Engineering, Ångström Laboratory, Uppsala University, Uppsala, Sweden.

[12] Subaru Telescope, 650 N Aohoku Pl, Hilo, HI 96720, USA.

[13] The Breakthrough Initiatives, NASA Research Park, Bld. 18, Moffett Field, CA, 94035, USA.

[14] Observatoire de Paris, 5 Place Jules Janssen, 92190 Meudon, France.

[15] California Institute of Technology, 1200 E. California Blvd., Pasadena, CA 91125, USA.

[16] Université Côte d'Azur, Observatoire de la Côte d'Azur, CNRS, Laboratoire Lagrange, Nice, France.

[17] Jet Propulsion Laboratory, California Institute of Technology, 4800 Oak Grove Dr., Pasadena, CA 91109, USA.

[18] Sterrewacht Leiden, Leiden University, Postbus 9513, 2300 RA Leiden, The Netherlands.

[19] Max Planck Institute for Extraterrestrial Physics, Giessenbachstraße 1, 85748 Garching, Germany.

* Correspondence to: kevinwagner@email.arizona.edu

This PDF file includes:

Supplementary Methods
Supplementary Figures 1 to 6
Supplementary Table 1
Supplementary References (*1–19*)

Supplementary Methods

Artifact Modeling I. Persistence Stripes

The most significant systematic artifact is detector persistence accumulated as a result of photons collected during the chopping sequence. These line-like features rotate with the field of view–resulting in reduced sensitivity throughout the entire image and greater false-positive potential in the areas directly between the stars. Either an empirical or a physically motivated model is needed to correct for this effect−similar to the charge trapping and delayed release models recently developed for HgCdTe detectors (e.g., *1, 2*). We constructed two persistence models, which are shown in Supplementary Fig. 1. The first is a simple empirical model that includes a superposition of lines convolved with the instrumental PSF. The second is a higher-fidelity model in which we simulated the chopping kinematics and charge accumulation. In this model, we assumed a persistence response that is proportional to the signal in the PSF and included a free parameter to scale the intensity of the persistence pattern of α Centauri B with respect to that of α Centauri A to remove the major contributions of non-linearity. We translated the PSFs of α Centauri A and B across a simulated image plane for both strokes of the chopping sequence, including a sub-pixel offset and corresponding re-centering behind the coronagraph at the end of each stroke. We do not account for a potential time decay of the signal and retain all accumulated intensity in the pattern. We subtracted the persistence model of the second stroke from that of the first, and then subtracted the combined model from the individual frames.

To fit the parameters, we measured the positions of the off-axis PSFs of α Centauri A and B in each individual image and performed a grid search with velocity (degenerate with intensity, which we did not vary) and the offset from the center of the coronagraph as free parameters. We assumed that the offset for the second stroke is opposite that of the first to mimic a small error in the chopping angle. For each set of parameters within the grid, we constructed and subtracted the persistence model from each individual image prior to PSF subtraction, and repeated the remaining steps of the data reduction–primarily including high-pass filtering and PSF subtraction via the KLIP algorithm. We adopted the parameters that minimized the standard deviation of pixel values within the final combined image in an annulus from 10−45 pixels (~0.45−2 arcsec).

Both models are able to qualitatively reproduce the persistence pattern observed in the data (see Supplementary Fig. 1). Following model subtraction, a greater speckle density is present in the areas covered by persistence. The speckle intensity distribution in this region is clearly non-uniform, with several maxima present primarily within the persistence pattern from α Centauri A. Therefore, we refrain from characterizing sources in the areas impacted by persistence (between the dashed lines in Fig. 4). Additionally, the residual noise in the areas not impacted by persistence is improved following subtraction of the simulated model prior to PSF subtraction. This is because the persistence rotates with the field of view and biases the PSF subtraction. Accordingly, we utilize the images generated by the removal of the simulated persistence model prior to high-pass filtering and PSF subtraction for the proceeding analysis. The detection and properties of the candidate C1 are not affected by the choice in persistence model (including without subtracting any model).

Artifact Modeling II. Optical Ghosts

The second most significant artifact that appears in the final images are two dark arcs−likely bright features in the off-axis PSF of α Centauri A−that come within 1" of the coronagraphic residual. These features are likely faint optical ghosts that are introduced by the spectral filter, and which

rotate with the parallactic angle to create arc-like features in the final image. The presence of these arcs weakens the overall image sensitivity. Similar to our handling of the detector persistence stripes, we created an empirical model of the dark arcs as circular segments of negative flux and subtracted this from the images before PSF subtraction. We repeated the process to optimize the parameters of the arcs: including radius, brightness, and width, while keeping the center fixed to the off-axis position of α Centauri A. Following the removal of the arcs, the image background within ~1" of the coronagraphic mask is nearly flat, with the exception of the persistence stripe residuals (see Supplementary Figs. 1–3).

Plate Scale and True North Calibration

We utilized the orbit of the binary as reported in (*3*) to calculate the instrumental plate scale and true North offset (from the parallactic angle zero-point) to be used for astrometric calibrations. In June 2019, the orbit fit predicts that α Centauri B should appear at ρ=5.15 arcsec and θ=−18.6º E of N with respect to α Centauri A. Based on this orbit fit and the apparent position of the stars in the median-combined, non-PSF subtracted images, we calculated a plate scale of 0.0456±0.0002 arcsec/pixel and a true North offset of −36.7º±0.1º East of North.

Background-Limited Sensitivity

To compute the background-limited sensitivity, we measured the standard deviation of pixel values in a region of the detector far from α Centauri A and B, and compared this to the flux measured in a four-pixel radius aperture centered on the off-axis PSF of α Centauri A. We converted this ratio to a 3-σ sensitivity estimate by multiplying by a factor of $(3 \times \sqrt{16\pi})$ to account for the aperture area, and arrived at 5×10^{-7} contrast with respect to α Centauri A, or about 65 µJy (the *N*-band flux of α Centauri A is 129 Jy, *4*). The background near the stars is higher (by a factor of two at 1 arcsec) due to the increased photon noise and the glow of the AGPM (*5*).

Simulated Planetary Contrast vs. Separation Curves

We first calculated the radiative equilibrium temperature of each simulated planet on a circular orbit as a function of its orbital semi-major axis (a), its Bond albedo (A_B), the stellar radius (R), and stellar temperature (T_{star}). This is given by Supplementary Equation (1), which is obtained by setting the planet's luminosity to be equivalent to its total input of irradiant energy from the star (*6*).

$$T_{\mathrm{eq}} = T_{\mathrm{star}}\sqrt{\frac{R}{2a}}(1 - A_{\mathrm{B}})^{1/4} \qquad (1)$$

We assumed T_{star} = 5800 K and R = 1.2 $R_\odot$ for A, and T_{star} = 5250 K and R = 0.86 $R_\odot$ for B (*48*). We then included additional heat as a fraction of the planet's equilibrium temperature via Supplementary Equation (2), and computed Blackbody spectra for the star and planet.

$$T_{\mathrm{planet}} = T_{\mathrm{eq}} * (1 + f_{\mathrm{extra}}) \qquad (2)$$

We then created simulated photometric measurements by integrating the spectra between 10–12.5 µm in steps of 0.1 µm. These synthetic photometric measurements were used to calculate planet-star contrast ratios. We repeated the analysis for planets of different radii, Bond albedo,

orbital semi-major axes, and additional heating conditions to compute the simulated contrasts that are utilized to estimate the properties of C1 and also for comparisons in Figs. 1, 4, & 5.

Despite its relative simplicity, this approach is sufficient to gain an understanding of the sensitivity of our single-band photometric measurements. Specifically, uncertainties in planetary properties (e.g., molecular content and sources of additional heating) preclude the usefulness of a more sophisticated model. Our model does not include a wavelength-dependent model of molecular absorption and emission processes. Instead, absorption processes are incorporated in bulk via the Bond albedo. For Earth's atmosphere, the dominant absorbers in the λ~8–13 µm range are O_3 at $\lambda < 10$ µm and CO_2 at $\lambda > 12.5$ µm (*7*). Therefore, the overall level of absorption for Earth-like planets is expected to be low within the λ = 10–12.5 µm range of the NEAR filter (*8*). For Neptune, the dominant feature within this range is C_2H_6 emission at 12.2 µm, which is an order of magnitude above the thermal continuum (*9*). For Jupiter, NH_3 absorption at ~11 µm is present at a comparable equivalent width to the also present C_2H_6 emission (*10*). Such planetary atmospheres at ~1 au would have comparable equilibrium temperatures to Earth, which will change the overall ratios of these constituents and their spectral contributions. However, more detailed modeling of such atmospheres also suggests that blackbody spectra are reasonable approximations for λ = 10–12.5 µm (*11*). This approach also does not account for specific processes that could be responsible for producing additional heating or non-thermal emission and includes the sum of all of these in a simple fractional parameter, f_{extra}. If the solar system's giant planets are an accurate indication of the larger population, then these sources of additional heating can be expected to raise the thermal infrared planetary fluxes by up to an order of magnitude compared to the predictions of radiative thermal equilibrium (corresponding to f_{extra}~0.8, which is above the maximum of f_{extra}~0.5 considered here; see *12, 13*).

While these models carry a significant uncertainty in the maximum luminosity, they establish useful lower limits in cases of low additional heat and Bond albedos representative of the solar system planets (A_B ~ 0.3 for Earth, *6*). Finally, we verified the predicted contrasts by comparing to the Earth's brightness at λ ~ 10 µm (*7*). A planet around α Centauri A would experience a similar irradiance as the Earth if it were at a distance of ~1.2 au, or ~0.9 arcsec. Scaled to a radius of 1.7 $R_\oplus$ and shifted to the distance of α Centauri (1.3 pc), such a planet would appear with a contrast of ~5×10^{-7}. Our corresponding model of a super-Earth with $f_{\text{extra}} = 0.1$, with the same planetary radius (1.7 $R_\oplus$), and at the same separation from the star (0.9 arcsec) has a contrast of ~4×10^{-7}. This serves as a useful consistency check of our models and assumptions.

Simulated Planet Injection and Retrieval Tests

To compute the image sensitivity, we performed extensive point source injection and recovery tests (see Figs. 3 & 4). For the injected PSF, we utilized the off-axis PSF of α Centauri A and included a scaling correction for the transmission of the AGPM (*14*). The PSFs were injected prior to high-pass filtering, after which we completed the remaining data processing steps (PSF subtraction, etc.). We measured the signal within an aperture of two pixels in diameter centered on the location of the injected source. This diameter corresponds to ~0.3×FWHM, which is smaller than typically used. This provides a larger number of apertures that are non-contaminated by persistence. To measure the noise, we blocked the regions of the image affected by detector persistence and a 12-pixel wide box centered on the injected source and calculated the mean and standard deviation of fluxes within all non-overlapping apertures at the same separation. We

converted these measurements into a signal-to-noise ratio (SNR) estimate (*15*), adopting SNR=3 as a detection threshold, as this resulted in all of the automatically retrieved sources also being identified by eye in the images (see Fig. 3). We caution that as a result of the residual structures in the images, the statistics are non-Gaussian, and probabilities derived from Gaussian statistics should not be applied to these measurements. We repeated the analysis with injected sources along ten different position angles and from separations of 0.3 to 1.5 arcsec. We adopted the average sensitivity level over the tested position angles as the sensitivity for each separation in the contrast curves shown in Fig. 4. For the completion analysis (see Fig. 5 and following subsection), we adopted the maximum brightness of the recovered sources as the sensitivity for each separation.

Completeness Analysis

We converted the sensitivity analysis into completion estimates using a Monte Carlo simulation to draw randomly sampled orbital parameters. For different sets of uniformly sampled planet radius and semi-major axis, we sampled the argument of periapsis ($\omega \propto$ unif[0 , 360]; this parameter is degenerate with the time of closest approach for the circular orbits considered here), angle of ascending node ($\Omega \propto$ unif[0 , 360]), and inclination ($i \propto \sin i$) and then determined the planet's separation and position angle on the sky. The radiative equilibrium temperature was determined assuming a Bond albedo of 0.3 and then increased by 10% and 50% to account for different amounts of internal/additional heat from the planet. The temperature was converted to an *N*-band contrast following the previous subsection. This contrast was then compared to the maximum point-source brightness at that separation that would have been detected based on simulated point-source injections (see above). The completeness maps are shown in Fig. 5. We also tested restricting the plane of the planetary orbits to the orbital plane of the binary, and also allowed for eccentric orbits, and found that these did not significantly alter the completeness maps.

Properties of The Candidate Detection 'C1'

Based on a similar analysis to the simulated planet injection and recovery tests, C1 is detected with *SNR*~3.1 (using apertures with diameter of two pixels, resulting in 27 noise measurements not contaminated by the persistence artifact). Using apertures with diameter of 1×FWHM, corresponding closely to the definition of *SNR* in (*15*), gives *SNR*~3.5, although with only ten independent noise measurements after rejecting seven due to persistence contamination. Again, we caution that probabilities derived from Gaussian statistics should not be applied to this *SNR* estimate. To measure the brightness and position of C1, we performed negative point source injections over a grid in radius, position angle, and contrast to minimize the residual squared flux in the total image area (see the bottom-left panel of Supplementary Fig. 4). We injected the point sources in the data processing prior to high-pass filtering and PSF subtraction and performed a similar analysis with both pipelines. The findings were consistent, and we averaged the results to arrive at $\rho = 0.85 \pm 0.05$ arcsec, θ=228.9º ±3.3º E of N, and $F_{C1}/F_{\alpha CenA} = 9.3 \times 10^{-6} \pm 3.1\times10^{-6}$, corresponding to F_{C1}=1.2±0.4 mJy, after correcting for the ~20% throughput of the coronagraph, and by estimating the astrometric uncertainties as the FWHM divided by the SNR. Assuming that the emission comes from a planet with a Bond albedo of ≤0.3 (similar to Earth), and that the planet's temperature is between 100−150% of its radiative equilibrium temperature (T_{eq}~300 K) to account for the possibility of additional sources of heat (such as greenhouse heating or residual heat from the planet's formation), and assuming blackbody spectra, this brightness and projected separation correspond to a radius of ~3.3−11 $R_\oplus$. This is a plausible radius and level of additional heating compared to the ice− and gas-giants within our own solar system (e.g., *12, 13*).

Pre-imaging for Background Sources

We first ruled out the possibility that C1 may be a background source by utilizing K-band (~2 μm) images taken in 2009 (*3*). The faintest source identifiable in these earlier images is a K~13.6 star that approaches the trajectory of α Centauri B in 2021. There is nothing comparable in brightness and proximity in mid-2019. Given that the NEAR campaign images are sensitive to N~12−12.5, and that even the reddest stars have neutral mid-to-near infrared colors, we can exclude the possibility that C1 is a background star. If C1 is a background galaxy, it would likely have a rest peak wavelength of $\lambda \sim 1$ μm and would thus be redshifted by $Z \sim 10$. Such a galaxy would be among the most distant galaxies observed, and unlikely to appear projected on the sky within an arcsecond of α Centauri A and with such an apparent brightness. In all of the HST legacy fields spanning >800 arcmin^2, only a handful of such galaxies were detected (*16*), and all have near-IR magnitudes >25, which is much fainter than our observations would have been able to detect.

Exozodiacal Dust Disk Modeling

We simulated an exozodiacal disk at the central observing wavelength of ~11.25 μm using *zodipic* (*17*). The code enables simulating both the disk and the star simultaneously, which, when convolved to the resolution of the observations, enables straightforward model flux calibration. We assumed a 79º inclination to be coplanar to the orbit of the binary (*3*) and fit the position angle and small central offset. Finally, we ran a grid search over the amount of dust in the model disk to match the brightness of C1. We subtracted each model from the data prior to high-pass filtering and PSF subtraction and adopted the model that minimized the squared residuals in an aperture of 10−45 pixels from the center as the best-fit. The results are shown in Supplementary Fig. 4 in comparison with the residuals from the point source subtraction.

Astrometry and Orbital Modeling

To measure the astrometry of C1, we combined back-to-back nights, and re-performed minimization of the image residuals by negative synthetic planet injection tests. The astrometric precision was estimated as 1/3 of a beam diameter, or ~8 mas. The measurements are shown in Supplementary Fig. 5. To check consistency of the measurements with orbital motion, we generated 10,000 sample orbits using the OFTI method (*18*) in the orbitize! package (*19*) with the default settings (i.e., uniform priors). One hundred randomly selected sample orbits are shown in Supplementary Fig. 5 along with the retrieved orbital parameters. The data are consistent with a co-planar and dynamically stable orbit interior to the α Centauri AB binary (a<3 au and i ~70º). While the astrometric data are consistent with a Keplerian orbit, we caution that this does not constitute proof of orbital motion, as the data are also consistent with no motion.

The measurements show a tentative change of $\Delta\rho \sim -0\ .1$ arcsec (i.e., toward the star) and ΔPA ~ 8º from 2019-05-24 to 2019-06-11, which establishes a useful vector to check against the data taken in late June. Projected forward in time, C1 would likely be at a separation of $\rho \sim 0.6{-}0.7$ arcsec on 2019 June 27. At this position, C1 would overlap the area of the image that is strongly impacted by the persistence stripes, and thus would not likely be detected. While C1 is not detected on this night, forward modelling of the anticipated signal assuming zero motion suggests that such a source would likely have been detected (see Supplementary Fig. 6). The non-detection of C1 on June 27, compared to its identification in ~85% of the single-night images taken prior to this date, tentatively supports the planetary hypothesis over those invoking exozodiacal dust or an unknown instrumental artifact, which would remain in a stable position throughout the campaign.

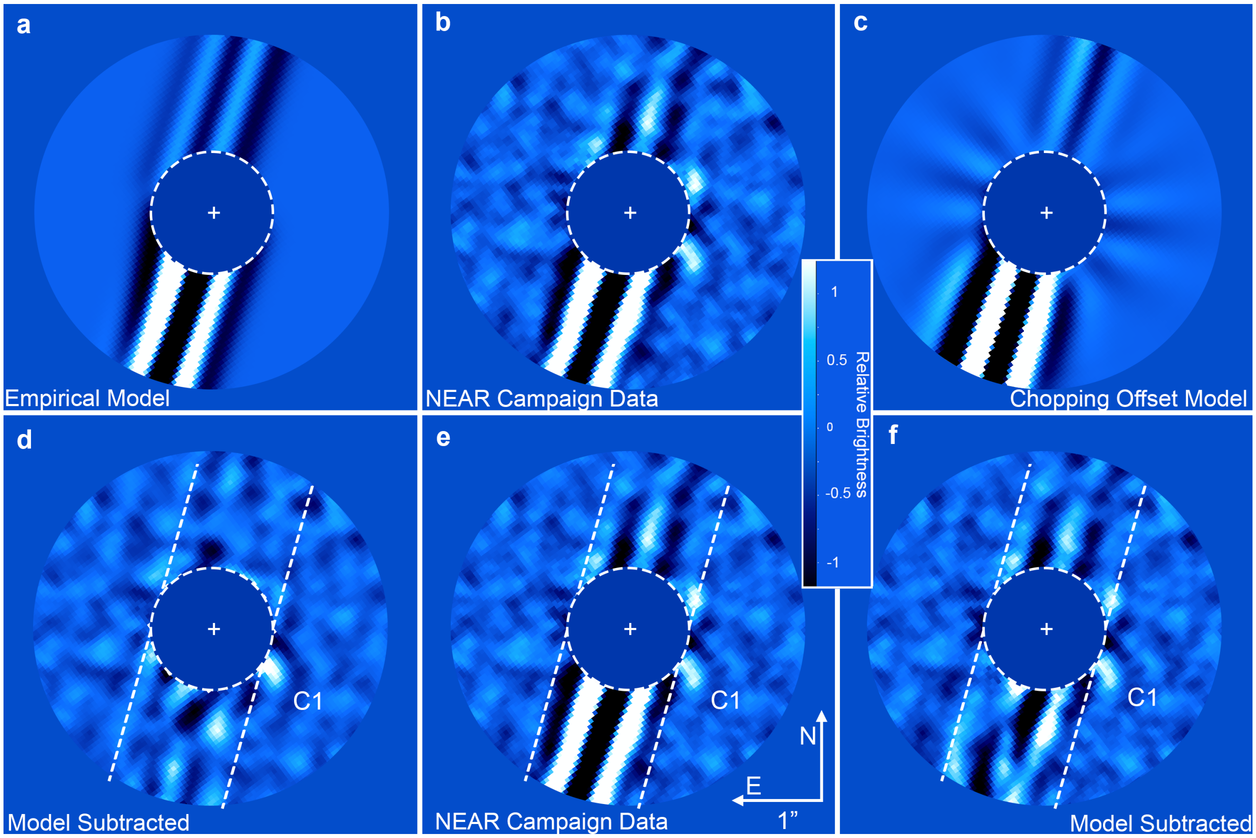

Supplementary Fig. 1. Detector persistence models and the NEAR campaign image following correction. The empirical model fit to the post-KLIP image (**a** and **d**) is subtracted after the KLIP procedure, while the simulated chopping offset model (**c** and **f**) is subtracted prior to the KLIP procedure. The central panels **b** and **e** show the original data without persistence model subtraction for comparison. The model image in **c** has been processed with KLIP to enable a straightforward comparison to **b** and **e**. Areas impacted by persistence are marked with dashed lines. We only consider sources exterior to these regions as potentially real detections. A point-like feature (C1) is marked as a candidate detection. Artifacts from optical ghosts have also been removed.

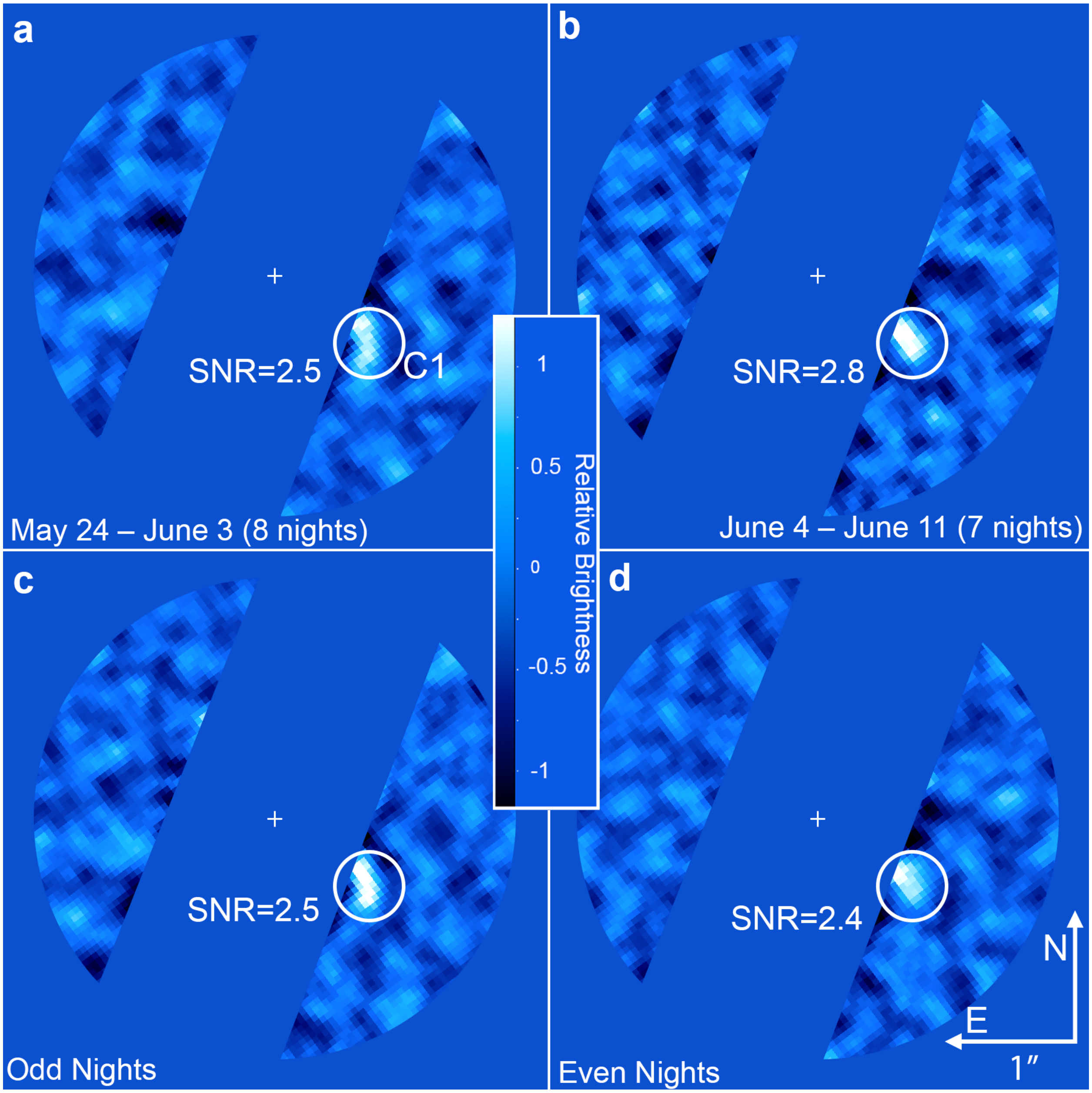

Supplementary Fig. 2. NEAR campaign data split into two sets of independent datasets. a & b compare the first and second halves of the campaign, respectively, while **c** & **d** compare even and odd nights. C1 is the only source clearly identified in each image outside of the area impacted by the persistence stripes (masked here).

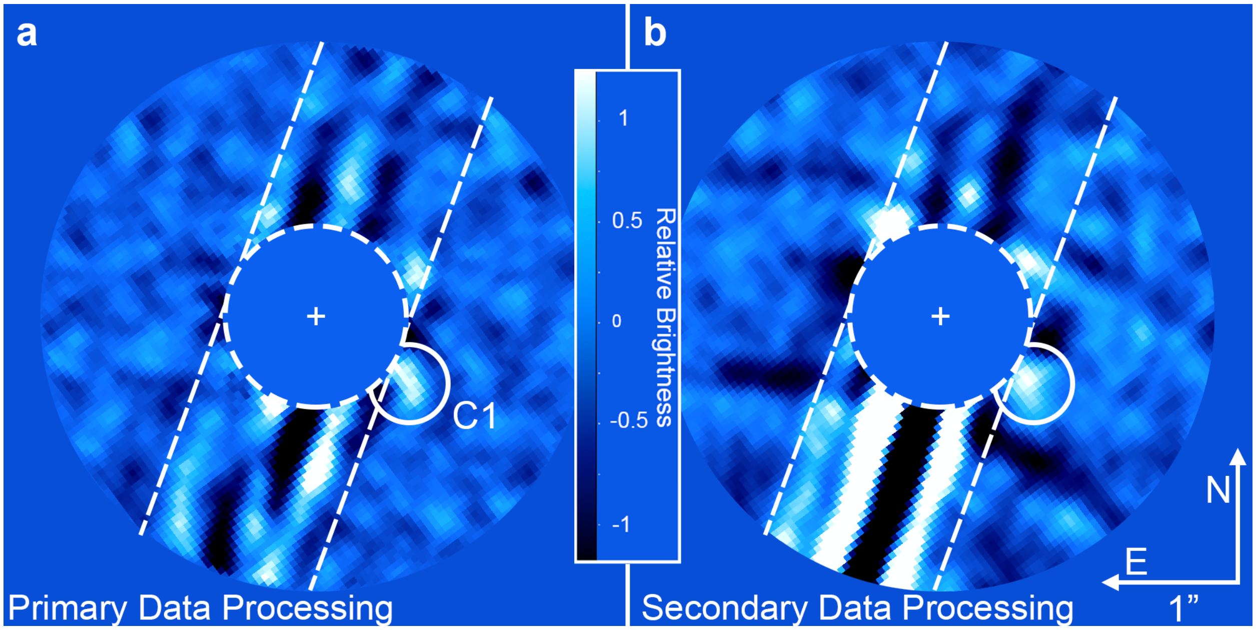

Supplementary Fig. 3. NEAR data processed by two completely independent data processing pipelines. The primary data processing (**a**) implements removal of the known systematic artifacts, whereas the persistence stripes and dark arcs are present in **b**. Excluding the known instrumental artifacts, C1 is the only source with a repeated detection in both images.

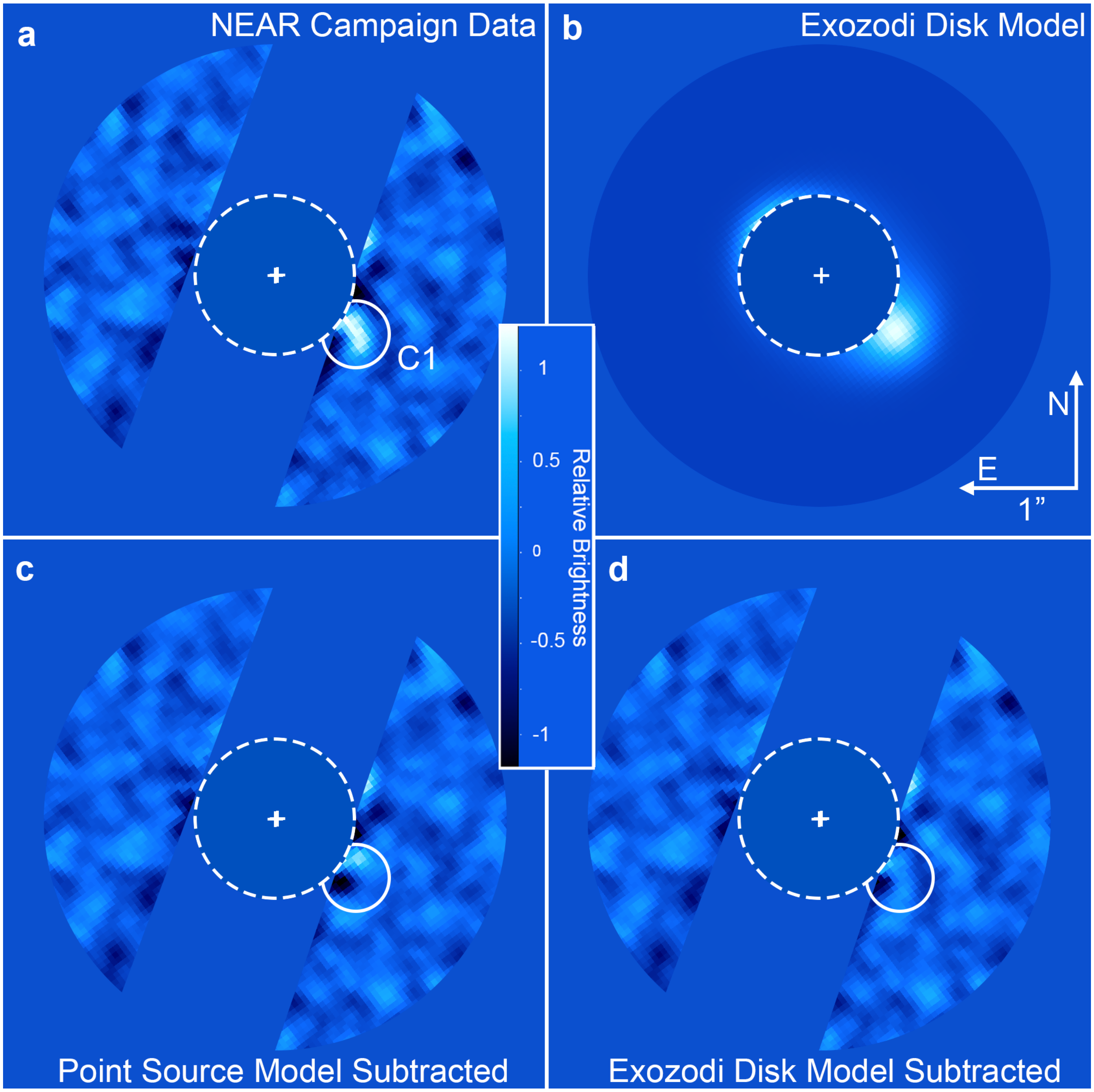

Supplementary Fig. 4. C1 subtracted via both an exoplanet and exozodiacal disk models. a shows the data without C1 subtracted for comparison. **c** shows the subtraction of C1 with a point source (i.e. exoplanet) model. **b** shows the model disk, which is inclined by 79º from the plane of the sky, extends from 0.8−1.2 au, includes 60 zodis of dust, and is offset by ~0.3 au in the direction of C1. **d** shows the image with the disk subtracted. Both models match the data reasonably well.

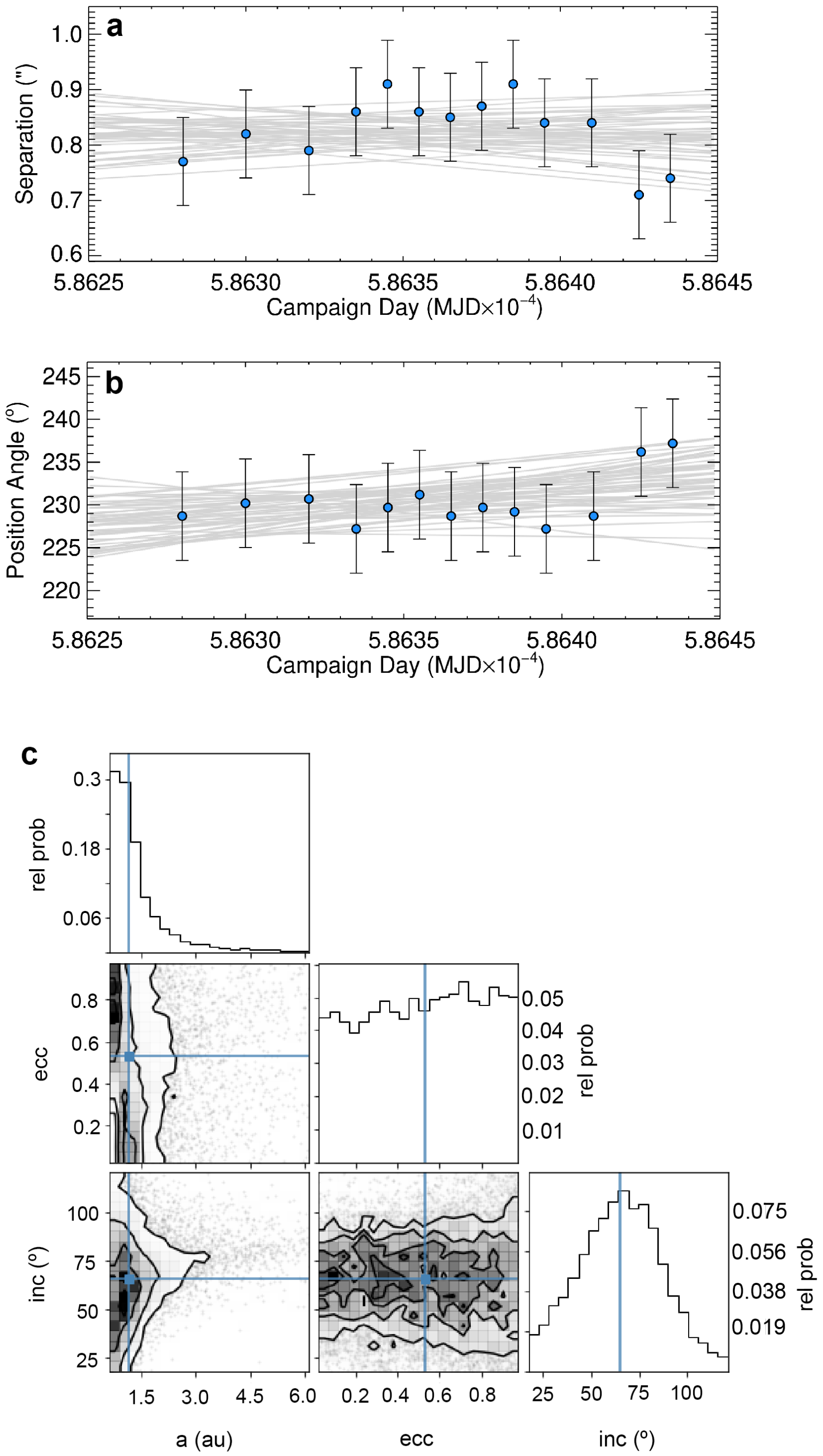

Supplementary Fig. 5. Astrometric data and orbital fit for C1 generated from the NEAR campaign. The astrometric data (**a, b**) are consistent with a bound orbit with a semi-major axis of ~1.1 au and an inclination of 65º±25º (**c**). Error bars in **a** and **b** represent the estimated astrometric uncertainty of 1/3 of a beam diameter, or ~8 mas. While the nature of the candidate is not yet certain, the consistency of a Keplerian orbit and its alignment with the orbit of the binary is a useful check for the hypothesis that C1 is a planet orbiting α Centauri A.

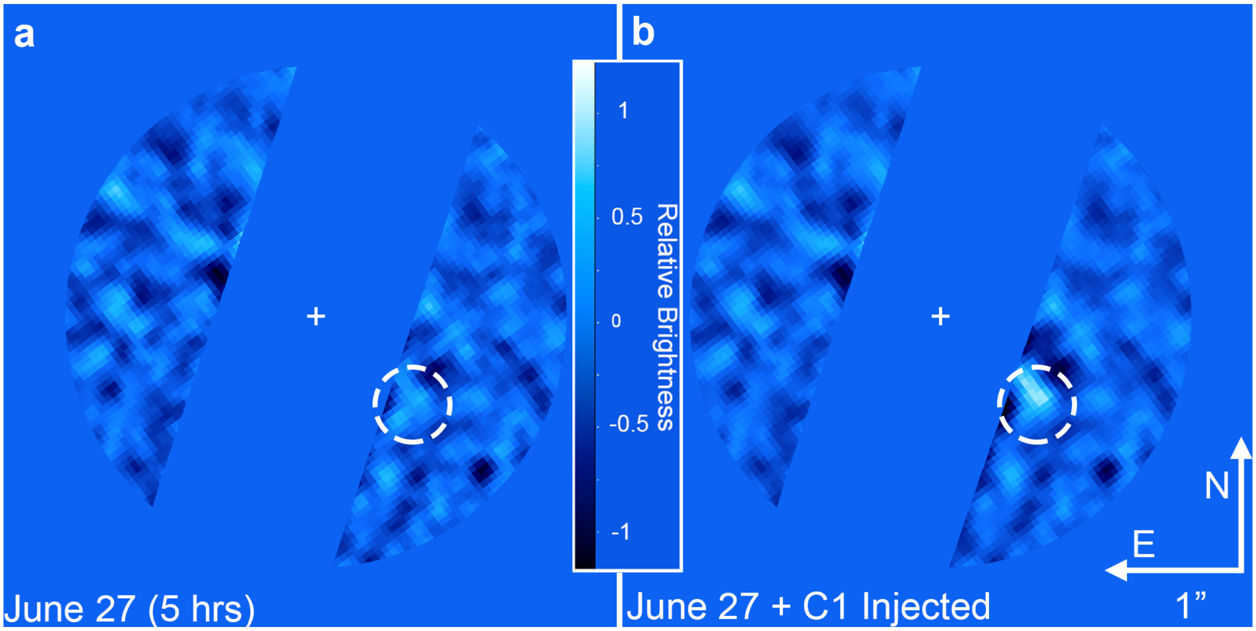

Supplementary Fig. 6. **Observations of α Centauri with NEAR on 2019 June 27.** The approximate position of C1 is indicated by the dashed circle. **a** shows the original data, and panel **b** shows the same data with a simulated planet with the properties of C1. These images illustrate that C1 would have likely resulted in a detection on June 27 if it remained stable in position and brightness.

UT Date 2019-	Int. Time (hr)	Avg. Seeing (“)	Ambient Temp (ºC)	PWV (mm)
5-24	6.3	1.0	10–12	<1
5-26	5.8	1.2	8–10	2.5–5
5-28	2.4	1.0	7–10	5
5-29	0.8	1.0	9–12	7.5
5-30	5.9	0.7	9–10	6.5
5-31	5.5	1.0	7–10	3.0
6-01	5.3	1.0	7–10	2.5–4
6-02	4.6	1.0	10	<1
6-03	6.4	0.7	15	2–2.5
6-04	6.3	0.7	17	2.5–3
6-05	5.4	1.0	17	1–2.5
6-06	3.3	1.2	14–16	1.25
6-08	2.7	0.5	12–17	1.1
6-09	5.9	0.7	15	1.25–2
6-10	3.1	0.5	12–17	2–2.25
6-11	2.2	1.5	12–14	2.5–3
6-27	5.0	1.2	9–10	2.5–5
	Total=76.9	Med.=1.0	Med.=11	Med.=2.8

Supplementary Table 1. Near Campaign Observing Log. Note: PWV = Precipitable Water Vapor. Seeing values correspond to differential image motion monitor (DIMM) measurements at λ~0.5 µm.

Supplementary References:

1. E. Agol, N. B. Cowan, H. A. Knutson, et al., The Climate of HD 189733b from Fourteen Transits and Eclipses Measured by Spitzer. *ApJ* ***721***, 1861 (2010).
2. Y. Zhou, D. Apai, B. W. P. Lew, et al., A Physical Model-based Correction for Charge Traps in the Hubble Space Telescope's Wide Field Camera 3 Near-IR Detector and Its Applications to Transiting Exoplanets and Brown Dwarfs. *AJ* ***153***, 243 (2107).
3. P. Kervella, F. Mignard, A. Mérand, et al., Close stellar conjunctions of α Centauri A and B until 2050 . An mK = 7.8 star may enter the Einstein ring of α Cen A in 2028. *A&A* ***594***, A107 (2016).
4. M. Cohen, R. G. Walker, M. J. Barlow, et al., Spectral Irradiance Calibration in the Infrared. I. Ground-Based and IRAS Broadband Calibrations. *AJ* ***104***, 1650 (1992).
5. O. Absil, D. Mawet, M. Karlsson, et al., Three years of harvest with the vector vortex coronagraph in the thermal infrared. *Proc. SPIE* ***9908***, 99080Q (2016).
6. I. de Pater, J. Lissauer, Planetary Sciences. Cambridge University Press, Cambridge, UK (2015).
7. D. Des Marais, M. Harwit, K. Jucks, et al., Remote Sensing of Planetary Properties and Biosignatures on Extrasolar Terrestrial Planets. *Astrobiology* ***2***, 153 (2002).
8. M. Kasper, R. Arsenault, H.-U. Käufl, et al., NEAR: Low-mass Planets in α Cen with VISIR. *The Messenger* ***169***, 16 (2017).
9. L. Fletcher, P. Drossart, M. Burgdorf, et al., Neptune's atmospheric composition from AKARI infared spectroscopy. *A&A* ***514***, 17 (2010).
10. L. Fletcher, T. Greathouse, G. Orton, et al., Mid-infrared mapping of Jupiter's temperatures, aerosol opacity and chemical distributions with IRTF/TEXES. *Icarus* ***278***, 128 (2016).
11. C. Beichman, M. Ygouf, J. Llop Sayson, et al., Searching for Planets Orbiting α Cen A with the James Webb Space Telescope. *PASP* ***132***, 015002 (2020).
12. J. C. Pearl, & B. J. Conrath, The albedo, effective temperature, and energy balance of Neptune, as determined from Voyager data. *J. Geophys. Res.* ***96***, 18921 (1991).
13. C. Reinhardt, A. Chau, J. Stadel, et al., Bifurcation in the history of Uranus and Neptune: the role of giant impacts. *MNRAS* ***492***, 5336 (2020).
14. A.-L. Maire, E. Huby, O. Absil, et al., Design, pointing control, and on-sky performance of the mid-infrared vortex coronagraph for the VLT/NEAR experiment. *JATIS* ***6***, 5003 (2020).
15. D. Mawet, J. Milli, Z. Wahhaj, et al., Fundamental Limitations of High Contrast Imaging Set by Small Sample Statistics. *ApJ* ***792***, 97 (2014).
16. P. A. Oesch, R. J. Bouwens, G. D. Illingworth, et al., The Dearth of z ~10 Galaxies in All HST Legacy Fields—The Rapid Evolution of the Galaxy Population in the First 500 Myr. *ApJ* ***855***, 105 (2018).
17. M. Kuchner, ZODIPIC: Zodiacal Cloud Image Synthesis. Astrophysics Source Code Library, 1202.002 (2012).

18. S. Blunt, E. L. Nielsen, R. J. De Rosa, et al., Orbits for the Impatient: A Bayesian Rejection-sampling Method for Quickly Fitting the Orbits of Long-period Exoplanets. *AJ* ***153***, 229 (2017).

19. S. Blunt, J. J. Wang, I. Angelo, et al., orbitize!: A Comprehensive Orbit-fitting Software Package for the High-contrast Imaging Community. *AJ* ***159***, 89 (2020).
